# Interactive Effects of Morphine on HIV Infection: Role in HIV-Associated Neurocognitive Disorder

**DOI:** 10.1155/2012/953678

**Published:** 2012-05-20

**Authors:** Pichili Vijaya Bhaskar Reddy, Sudheesh Pilakka-Kanthikeel, Shailendra K. Saxena, Zainulabedin Saiyed, Madhavan P. N. Nair

**Affiliations:** ^1^Department of Immunology, Institute of NeuroImmune Pharmacology, Herbert Wertheim College of Medicine, Florida International University, Miami, FL 33199, USA; ^2^Centre for Cellular & Molecular Biology, Uppal Road, Hyderabad 500007, India

## Abstract

HIV epidemic continues to be a severe public health problem and concern within USA and across the globe with about 33 million people infected with HIV. The frequency of drug abuse among HIV infected patients is rapidly increasing and is another major issue since injection drug users are at a greater risk of developing HIV associated neurocognitive dysfunctions compared to non-drug users infected with HIV. Brain is a major target for many of the recreational drugs and HIV. Evidences suggest that opiate drug abuse is a risk factor in HIV infection, neural dysfunction and progression to AIDS. The information available on the role of morphine as a cofactor in the neuropathogenesis of HIV is scanty. This review summarizes the results that help in understanding the role of morphine use in HIV infection and neural dysfunction. Studies show that morphine enhances HIV-1 infection by suppressing IL-8, downregulating chemokines with reciprocal upregulation of HIV coreceptors. Morphine also activates MAPK signaling and downregulates cAMP response element-binding protein (CREB). Better understanding on the role of morphine in HIV infection and mechanisms through which morphine mediates its effects may help in devising novel therapeutic strategies against HIV-1 infection in opiate using HIV-infected population.

## 1. Introduction

The HIV epidemic continues to be the most severe public health problem and concern within USA and across the globe with about 33 million people infected with HIV.During the later stages of the disease, HIV-1-infected patients suffer from a wide range of neurological and neurocognitive disorders collectively known as HIV-associated neurocognitive disorder (HAND) [1–6]). Severe neuropathological changes resulting in significantly higher neurocognitive dysfunctions have been linked with other infections or illicit drug abuse. According to recent studies, it is believed that illicit drug abuse is one of the leading causes for transmission of HIV within USA [7–13]. Injection drug users are at a higher risk of getting infected with HIV and have greater chances of developing neurological abnormalities and other opportunistic infections as a result of sharing contaminated needles and increased risky sexual behavior [14–16]. Combined HIV infection along with opiate drug addiction has gained attention in the recent years and is an emerging problem in the post-HAART era since these individuals live longer; however, the associated neurological abnormalities remain among most of the clinical disorders observed in HIV-infected patients [8–10, 12, 17, 18].

Opioids represent a major class of addictive drugs of abuse among which heroin is the most abused substance. Since heroin is converted to morphine in the brain, morphine has been the preferred choice of study [19]. Morphine has been demonstrated to severely affect the immune system by modulating the functions of variety of cells like phagocytes, T cells, and dendritic cells [20–24].

Opioid drugs and HIV viral proteins act in synergy and thus lead to greater immunosuppression and hence these drugs are called cofactors for HIV infection. Selective regions of the brain such as striatum and hippocampus highly express opioid receptors and have been associated with increased viral titers in HIV-infected patients [19, 25, 26]. Apart from modifying the neural response to HIV directly, opiate drugs also affect the endogenous opioid peptide levels which in turn modulate the functions of the central nervous system [19, 25]. Though different kinds of opioid receptors exist, the commonly used opioids such as morphine and opioid agonists like naloxone bind to the *μ* opioid receptor with greatest affinity. This paper summarizes the results from our and other laboratories supporting the hypothesis that morphine enhances HIV-1 infectivity and that it aids in the neuropathogenesis of HAND.

## 2. Morphine Induces Apoptosis

**Figure 1:**
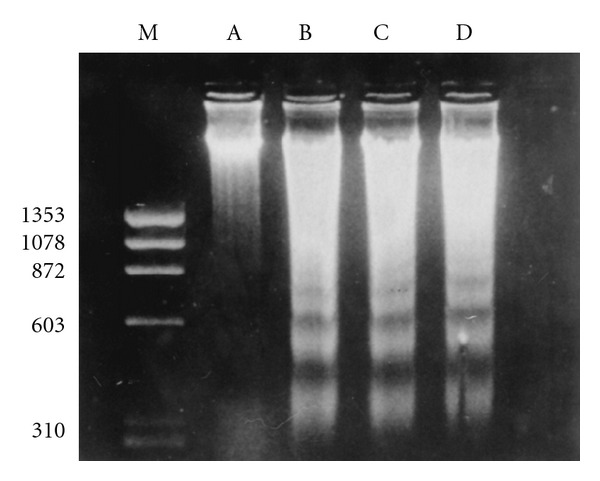
Morphine induces apoptosis of human PBMCs. PBMCs were cultured alone or with different concentrations of morphine for 60 h. Total DNA was extracted and electrophoresed on a 1.8% agarose gel in the presence of ethidium bromide. Arrows indicate fragmented DNA. Lane A, control culture; lanes B and C, cells treated with morphine at concentrations of 10^−7^ and 10^−9 ^M respectively; lane D, cells treated with cortisol at 0.2 mg/mL (positive control); lane M, molecular weight marker. Copyright © 1997, American Society for Microbiology. This figure is reproduced from the original article published in Clinical and diagnostic laboratory immunology, Nair et al. [35].

Chronic abuse of opiate drugs significantly increases the viral titers and affects the CD4 T cells in HIV-infected subjects [27, 28]. Apoptosis has been postulated to be a cause for the significant loss of T cells, thereby worsening the clinical condition of HIV-infected patients. HIV virus and many of the proteins that are encoded by HIV genome including gp120, Tat, Nef, Vpr, Vpu, and HIV protease have been found to have pro- and/or antiapoptotic qualities [29–32]. Peterson and colleagues have shown that morphine increases HIV-1 replication in human peripheral blood mononuclear cells (PBMCs) that are chronically infected with HIV [33, 34]. Our studies for the first time have indicated that PBMCs treated with morphine induce significant apoptosis (Figure 1). Cells treated with morphine showed evident DNA fragmentation as compared to their respective controls. Our studies in total suggested that morphine can cause immunosuppression during HIV infection possibly by inducing apoptosis either independently or by acting as a cofactor in the pathogenesis of HIV infection [35]. In another study, Moorman et al. [36] reported that freshly isolated human PBMCs when infected with either HIV-1 gp120/anti-gp120 or morphine (3 *μ*M) alone did not cause a significant apoptosis. However, a combined infection with HIV-1 gp120/anti-gp120 in the presence of morphine significantly enhanced the percentage of apoptotic cells. Opiate drugs bind to *μ* opioid receptors present on different kinds of immune cells and thus affect the inflammatory responses including macrophage modulation and production of different kinds of cytokines [33, 37, 38]. Upregulation of *μ* opioid receptor has been suggested to play an important role in HIV infection [39]. In correlation with these results, studies of Moorman et al. have demonstrated that mice lacking *μ* opioid receptors showed significantly lowered apoptosis as compared to wild types when treated with HIV-1 gp120 and morphine [36].

Although morphine primarily exerts its effect by binding to the *μ* opioid receptors, it also can bind to other receptors such as kappa and delta. Studies have shown that treatment of CD4^+^ T lymphocytes with synthetic kappa opioid receptor (KOR) ligands significantly suppressed HIV p24 and CXCR4 expression and such inhibitory action of the KOR ligand was further shown to be operative in the initial phase of the virus entry into the cell [40]. In addition, pretreatment of CD4^+^ lymphocytes with U50,488 inhibited HIV-1 envelope glycoprotein-mediated membrane fusion in a dose-dependent manner and such inhibitory activity was blocked by the KOR antagonist nor-binaltorphimine [41].

Morphine can cross the blood brain barrier (BBB) causing cerebral dysfunction. Recent studies have demonstrated that morphine induces apoptosis in human neurons and such effect was further enhanced in combination with HIV [42–44]. Further, studies have shown that morphine enhances HIV-Tat-induced toxicity in human neurons through the opioid receptors by increasing caspase-3 activity and decreasing BCl2/BAX ratio indicating that the cells progressed towards apoptosis [42, 44]. Further, addressing the combined effects of morphine and HIV, Hauser et al. [42] have demonstrated that Tat alone or morphine alone resulted in an enhanced immunochemical expression of the active caspase-3 in young oligodendrocytes *in vitro*. However, the expression of caspase-3 was much higher and additive when exposed to Tat and morphine together and such upregulation was specifically blocked by the opioid antagonist naloxone. Further investigations employing the transgenic mice conditionally expressing HIV-Tat in the astroglia revealed that Tat and morphine showed additive effects in oligodendrocytes [42]. 

P38 MAPK has been shown to be involved in HIV-induced neuronal apoptosis [45]. The mechanisms involved in mediating the synergistic effect of HIV and morphine have also been shown to involve p38 MAPK [43, 45, 46]. In support of this hypothesis, pretreatment with SB203580, an inhibitor of MAPK, significantly attenuated the synergistic effect of HIV viral proteins and morphine. Further, phosphorylation of p38 MAPK by either morphine or HIV gp120 alone was short lived as compared to a combined treatment with morphine and HIV-1gp120 [43].

## 3. Morphine Alters the Cytokine Expression in Human Glial Cells

HIV is known to infect various CNS cells like microglia, astrocytes and cause neuronal dysfunction leading to dementia. Astrocytes represent a major population of nonneuronal cells in the brain that comprise about 25–50% of the total volume of the brain. Several studies have shown the important role played by astrocytes in supporting the neurons and their function. Although astrocytes have been shown to play a vital role in the neuropathogenesis of HIV, astrocytes are not productively infected with HIV unlike other cells such as macrophages, microglia, and monocytes.

**Figure 2:**
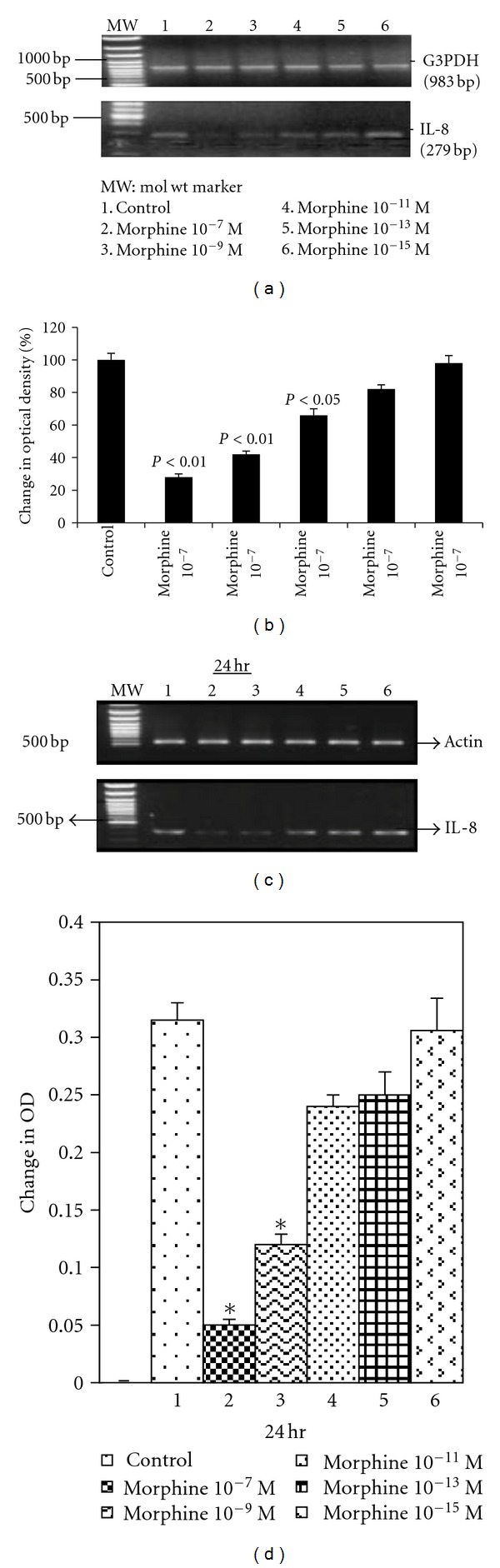
Morphine inhibits IL-8 gene expression in astrocytes and U87 astrocytoma cells. 3 × 10^6^ cells were cultured and treated with or without morphine for 24 h. RNA was extracted, reverse-transcribed, and amplified by PCR using primers for IL-8 and housekeeping gene G3PDH. PCR products were resolved on 1.2% agarose gel by electrophoresis. G3PDH remained unchanged at all periods while morphine significantly suppressed the expression of IL-8 gene in a dose-dependent manner in U87 astrocytoma (a). Quantitation of the effect of morphine on IL-8 gene expression by densitometry in U87 cells after normalizing to housekeeping gene (b). Kinetics of morphine inhibition of IL-8 gene expression in human astrocytes treated with different doses of morphine for 24 h (c). Quantitation of the effect of morphine on IL-8 gene expression in astrocyte cultures by densitometry after normalizing to housekeeping gene (d). This figure is reproduced from the original article in Journal of Immunology, 169: 3589-99, Mahajan et al. [47]. Morphine regulates gene expression of alpha- and beta-chemokines and their receptors on astroglial cells via the opioid mu receptor. Copyright 2002. The American Association of Immunologists.

Morphine is a potential immunosuppressant that crosses the BBB and regulates immune responses by different mechanisms in the central nervous system. Neuroinflammation is a hallmark of HAND associated with changes in chemokine expression. As said earlier, CNS may be specifically susceptible to synergistic effects of opiate abuse and HIV infection [47–49]. *μ* opioid receptor has been shown to be expressed in astrocytes which may be responsible for the increased effects of opiate drugs on HIV infection mediated by mechanisms such as increased calcium and altered cytokine productions [50]. Astrocytes get activated as a result of the alteration in the cellular homeostasis thereby leading to the production of cytokines [51, 52]. IL-8 is one of the first proinflammatory chemokines that was identified and responds in combination with other inflammatory factors [53–56]. IL-8 is known to inhibit the HIV infection by blocking its specific receptor CXCR2 [57, 58]. Our studies have shown that morphine at concentrations of 10^−7 ^M (*P* < 0.01), 10^−9 ^M, 10^−11 ^M significantly decreased the IL-8 expression in U87 astrocytoma cell lines by 29% (*P* < 0.01), 47% (*P* < 0.01), and 68% (*P* < 0.05), respectively (Figure 2), suggesting that morphine acts as a cofactor for the HIV infection leading to a significant down-regulation of IL-8 gene expression in a dose-dependent manner. Similarly, morphine suppressed IL-8 gene expression in primary astrocyte cultures [47]. Further, treatment of U87 cell lines and human astrocyte cultures with morphine for 24 h significantly inhibited the synthesis and secretion of IL-8 protein [47]. Down regulation of IL-8 by morphine was completely blocked by the **μ** receptor antagonist, *β*-funaltrexamine suggesting that the suppression of IL-8 is mediated through this opioid receptor.

Previous studies show that morphine also modulates the expression of other cytokines such as IL-6, IL-1*β*, and TNF-*α* during HIV infection. [9, 59]. IL-6 and IL-1*β*, expression was twice higher in HIV Tat and morphine-treated BV2 microglial cells (40-fold) as compared to the Tat-only-treated cells (20-fold). Similarly, TNF-*α* expression was at least twofold greater in the HIV Tat- and morphine-treated cells than those treated with Tat alone suggesting that morphine exacerbates the cytokine expression. Consistent with these results Bhokari et al. have reported similar changes in the cytokine expression associated with primary mouse microglial cultures [9]. In addition to these cytokines, an increased expression of IL-12 has been reported in astrocytes upon HIV and morphine exposure; however the increase in the IL-12 was not as prominent and as consistent as the other cytokines [59]. In another study, HIV Tat has been shown to potentiate the release of neuroactive cytokines in microglial cells [60]. Studies of El-Hage et al. [50] have further identified that the combined effects of HIV and morphine are modulated by the activation of the transcription factor NF-*κ*B which in turn is mediated by elevated intracellular calcium levels. Consistent with these results HIV Tat and morphine together increased IkB*α* phosphorylation and induced p65 translocation into the nucleus. Interestingly, one study has demonstrated that the cotreatment with morphine significantly inhibited Tat-induced cytokine production in N9 murine microglial cells suggesting the immunosuppressive role of morphine [61]. The reason for a decrease in the IL-8 and an increase in other cytokines like TNF-*α*, IL-6, and IL-1*β* is not clear. Detailed studies may be warranted to delineate these differential effects. 

## 4. Morphine Decreases the ***β***-Chemokine Expression in Astrocytes

Chemokines have taken a central focus in the recent years mainly due to the fact that they possess inhibitory effects on HIV infection [62, 63]. Chemokines represent small proteins of 5–12 kDA in size that are known to act as chemoattractants for NK cells, T cells, monocytes, neutrophils, fibroblasts, and endothelial cells. They are important mediators of transmigration of leucocytes across the BBB and play a significant role in the neuropathogenesis of HAND since these molecules recruit and regulate the movement of inflammatory cells into the CNS. Further, chemokines also regulate the degree of HIV infection [64, 65]. Deregulation in chemokine expression is a common phenomenon that is associated with astrocytes, microglia, macrophages, neurons, and endothelial cells exposed to virus, viral proteins and also in HIV dementia patients [66–69]. MIP-1*β*, also known as macrophage inflammatory protein has been reported to block CCR5 and CCR3 receptors [58, 70, 71]. Studies from our laboratory have shown that astrocytes treated with morphine at concentrations 10^−7 ^M, 10^−9 ^M, 10^−11 ^M for a period of 48 h significantly inhibited the protective gene MIP-1*β* expression by 78%, 65%, and 43%, respectively [49]. Monocyte chemoattractant protein (MCP)-1 is another CC-chemokine ligand (CCL2) that is particularly important in HIV neuropathogenesis in part because it mediates mononuclear phagocytes and leukocytes migration into the brain [66, 67]. Lines of evidence have indicated that the levels of CCL2 (MCP-1) and the CCR2 receptor levels very well correlate with the neurocognitive defects accompanying the neuropathogenesis of HIV disease progression [72, 73]. Earlier studies have shown that exposure of astrocytes with HIV-Tat and morphine together result in a synergistic increase in the release of the CCL2; however such synergism was lacking in the microglial cells [59]. Constitutively expressed CCL2 mRNA was significantly upregulated in human neurons by morphine exposure in a concentration and time-dependent manner but not in human astrocytes and microglia cells even though microglia and astrocytes also constitutively express CCL2 [74]. A *μ* opioid receptor agonist [D-Ala2, N-Me-Phe4, Glyol5] enkephalin (DAMGO) was shown to increase the expression of proinflammatory chemokines like CCL2, CCL5, and CXCL10 in PHA-stimulated human PBMCs at both protein and mRNA levels [75]. Increased CCL2 in astrocytes near the area of HIV Tat injection following the systemic administration of morphine indicates the role of morphine in enhancing inflammatory responses [48]. Consistent with these reports, it has been shown that glial activation and inflammation was attenuated completely in the CCR2 knockout mice compared to the wild type after the Tat and morphine injection either alone or together [76]. In addition to CCL2, CCL5, and CCL3 (MIP-1*α*) protein and mRNA expression levels were significantly exacerbated when exposed to Tat and morphine together as compared to either of them when present alone. Further these studies indicated that the ability of morphine to enhance the Tat-induced chemokine production is mediated by *μ* opioid receptors [59].

## 5. Effect of Morphine on Chemokine Receptors

Infection of cells with HIV requires the presence of coreceptors like CCR5, CCR2b, and CCR3 in addition to CD4^+^ receptors [58, 70]. These coreceptors are located in various cell types including brain cells [77]. It is possible that chemokines bind to more than one specific receptor. Mip-1*β* binds to CCR5 and CCR3 while MCP-1 binds to the CCR2b receptor. Both chemokines and their receptors have been demonstrated to play important roles in the neuropathogenesis of HIV infection. These receptors have also been shown to be present in higher levels in autopsied brain samples obtained from AIDS patients and have been shown to act as coreceptors for HIV infection [78–83].

**Figure 3:**
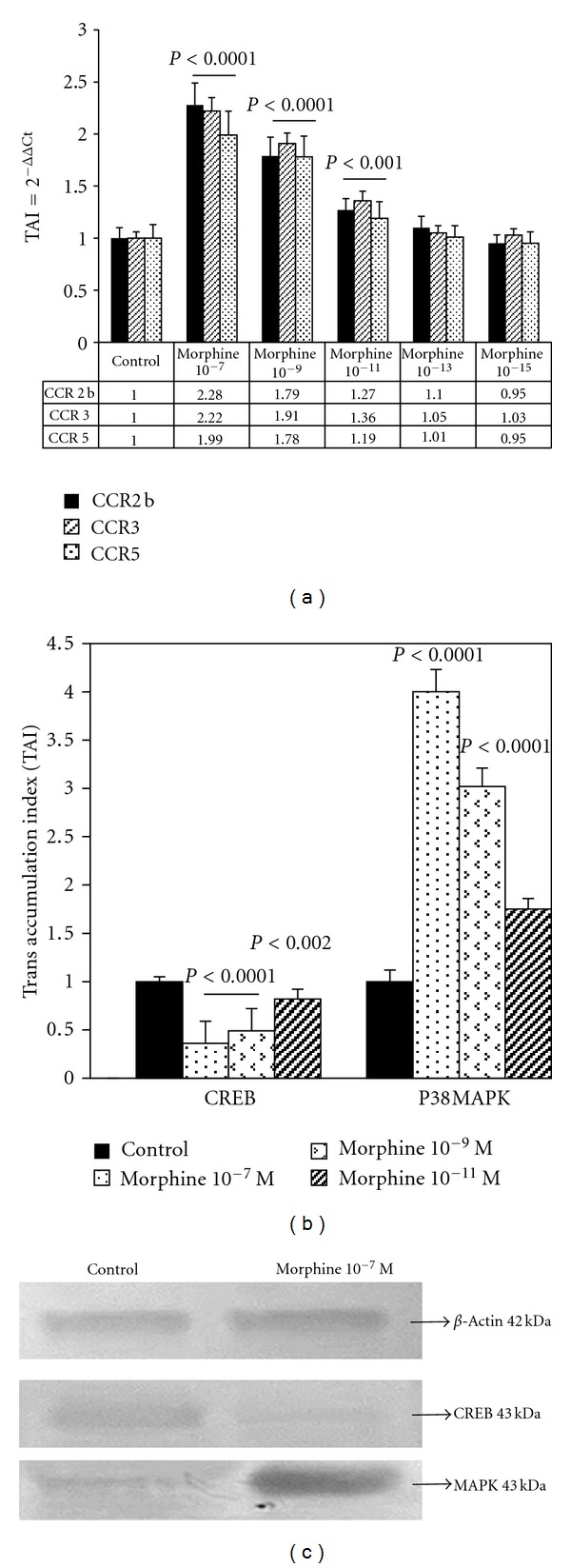
Morphine enhances the expression of CCR2b, CCR5, and CCR3 genes in human astrocytes (a). Astrocyte cultures were treated with different concentrations of morphine for 48 h and the relative expression of the mRNA was determined by real-time PCR. Morphine significantly upregulated p38 MAPK and downregulated CREB gene (b) and protein expression in astrocytes (c). This figure is reproduced from Clinical Immunology, 115: 323-32, Mahajan et al. [49, 84]. Morphine modulates chemokine gene regulation in normal human astrocytes. Copyright 2005, with permission from Elsevier.

CC-chemokine ligand 5 (CCL5) also known as RANTES specifically attracts and aids in the migration of the mononuclear macrophages and leukocytes to the site of infection [85]. CCL5 is known to preferentially stimulate an important HIV-receptor CCR5. Activation of CCR5 regulates the pathogenesis of HIV and simian immunodeficiency virus infection [86–88]. El-Hage et al. [59] have reported an increase in the CCL5 in HIV Tat-exposed astrocytes. In addition, morphine increases the CCR5 expression in astrocytes [49]. Further, the inflammatory and toxic effects of Tat and morphine together were attenuated in CCL5 null mice [86]. Our studies have demonstrated a dose-dependent increase in HIV-1 coreceptors CCR2b, CCR3, and CCR5 in astrocytes treated with morphine [49] (Figure 3(a)). Further, the addition of opioid receptor antagonists, naloxone *β*-funaltrexamine, to astrocyte cultures reversed the morphine-induced effects suggesting that the morphine-mediated effects were via *μ* opioid receptor [49]. Mahajan et al. have also shown that HIV-1 gp120-induced chemokine receptor expression is exacerbated by morphine in U773 astrocytoma cell lines [84]. Studies by other investigators have also confirmed the finding that morphine enhances HIV infection by significantly upregulating CCR5 and such increase was associated with inhibition of the endogenous production of beta chemokines in human mononuclear phagocytes [89]. In addition to these studies, morphine has been shown to upregulate CCR5 in CD3^+^ lymphoblasts and CD14^+^ monocytes [90]. In another study, Bokhari and colleagues have demonstrated the upregulation of the CCR5 in the murine microglia when treated with morphine and such effect was abolished by treatment with an opioid receptor antagonist naloxone [9]. Together these studies indicate that morphine plays a positive role as a cofactor in the neuropathogenesis of HAND.

## 6. Signal Transduction Mechanisms That Mediate-Morphine-Induced Effects in HIV-1

Changes in the expression of chemokines and their receptors can lead to signaling events that regulate various biological responses such as increased calcium influx and mitogen-activated protein kinase (MAPK) activation. Endogenous opioid peptides and agonists of opioid drugs have the potential to activate various signaling cascades either by suppressing adenyl cyclase, cation channels, or MAPK pathways [91]. Studies from our laboratory have shown that morphine increases p38 MAPK and down-regulates cAMP response element binding (CREB) gene and protein expression in U87 astrocytes [49] (Figures 3(b) and 3(c)). Consistent with these studies, other investigators have identified the involvement of various mitogen-activated kinases in morphine-mediated toxicity [92, 93]. Stimulation of MAPKs has been shown to regulate the HIV-1 infectivity [94–96]. Binding of morphine to the *μ* opioid receptor stimulates G-protein-associated molecules thereby inhibiting cAMP and resulting in lowered phosphorylation of CREB [91]. CREB mediated transcription is an important factor in neuronal adaptive response and has been demonstrated to be vital for normal neurocognitive functioning [97–99]. Increased phosphorylation of ERK1/2, JNK, p38, and Akt in a time-dependent manner has been reported in human brain microvascular endothelial cells when exposed to morphine [100]. In another study using human neurons, Malik et al. showed that the synergistic effects of HIV Tat and morphine involve JNK and ERK1/2 pathways; however, there were no changes in the p38 activation [44]. Chronic treatment of activated T cells with morphine inhibited the phosphorylation and activation of ERK1/2 and p38 MAPK further leading to a downregulation of the transcription factors activator protein-1 (AP-1), nuclear factor of activated cells (NFAT) and NF*κ*B [101].

Platelet-derived growth factor has been reported to be a mitogen and chemoattractant for different types of cells *in vivo* and *in vitro* and have the ability to induce the production of cytokines [102–104]. PDGF has been identified to be expressed at a higher level in the brain of macaques with SIV encephalitis [105]. Recent reports have demonstrated that human brain microvascular endothelial cells (HBMECs) exposed to morphine show increased PDGF mRNA as well as protein expression, leading to the activation of its downstream target transcription factor, namely, early growth response-1(Egr-1) [100]. The activation of both PDGF and Egr-1 was blocked by using specific inhibitors for ERK, JNK, and P38 indicating the role of MAP kinases in the PDGF activation [100]. Further, blockade of the *μ* opioid receptor by its antagonist naloxone significantly reduced the upregulation of both PDGF and Egr-1 suggesting that these events are mediated through the opioid receptor. The studies of Wen et al. further show that morphine activates Akt pathway; however this molecule does not mediate the activation of either PDGF or Egr-1 [100]. Activation of PDGF has been reported to be involved in BBB damage. Down regulation of ZO-1, a tight junction protein by morphine, has also been reported to be mediated by the *μ* opioid receptor and PDGF [106, 107].

## 7. Conclusions

These studies suggest that the drugs of abuse such as morphine enhance HIV-1 replication and infectivity in various cell types such as PBMCs and CNS cells. The mechanisms involved in such effects may be mediated by altering HIV-1 coreceptors and chemokines. The ability of opioids to alter the expression of chemokines and chemokine receptors by various cells of the central nervous system may significantly enhance the ability of HIV to infect the brain. Morphine also alters the status of both pro- and antiapoptotic molecules finally leading to a higher rate of apoptosis which is further exacerbated in case of HIV infection and such events are mediated by various signaling mechanisms. Overall, HIV infection in the brain may be enhanced either by viral binding and cellular uptake by upregulating HIV coreceptors or chemokine expression. Together, these studies provide important information on the molecular aspects of morphine, HIV-1 infection, and HIV pathogenesis, which may help in developing novel anti-HIV strategies targeting the coreceptors and chemokines.
